# Improving the classification of veterinary thoracic radiographs through inter-species and inter-pathology self-supervised pre-training of deep learning models

**DOI:** 10.1038/s41598-023-46345-z

**Published:** 2023-11-09

**Authors:** Weronika Celniak, Marek Wodziński, Artur Jurgas, Silvia Burti, Alessandro Zotti, Manfredo Atzori, Henning Müller, Tommaso Banzato

**Affiliations:** 1 University of Applied Sciences Western Switzerland (HES-SO), 3960 Sierre, Switzerland; 2 Faculty of Electrical Engineering, Automatics, Computer Science and Biomedical Engineering, AGH University of Krakow, 30059 Kraków, Poland; 3 Department of Animal Medicine, Productions, and Health, Legnaro (PD), University of Padua, 35020 Padua, Italy; 4 Department of Neuroscience, University of Padua, 35121 Padua, IT Italy; 5 Medical Faculty, University of Geneva, 1206 Geneva, Switzerland; 6 The Sense Research and Innovation Insitute, 1950 Sion, Switzerland; 7 Padova Neuroscience Center, University of Padova, Via Orus 2/B, 35129 Padova, Italy

**Keywords:** Machine learning, Animal physiology, Biomedical engineering

## Abstract

The analysis of veterinary radiographic imaging data is an essential step in the diagnosis of many thoracic lesions. Given the limited time that physicians can devote to a single patient, it would be valuable to implement an automated system to help clinicians make faster but still accurate diagnoses. Currently, most of such systems are based on supervised deep learning approaches. However, the problem with these solutions is that they need a large database of labeled data. Access to such data is often limited, as it requires a great investment of both time and money. Therefore, in this work we present a solution that allows higher classification scores to be obtained using knowledge transfer from inter-species and inter-pathology self-supervised learning methods. Before training the network for classification, pretraining of the model was performed using self-supervised learning approaches on publicly available unlabeled radiographic data of human and dog images, which allowed substantially increasing the number of images for this phase. The self-supervised learning approaches included the Beta Variational Autoencoder, the Soft-Introspective Variational Autoencoder, and a Simple Framework for Contrastive Learning of Visual Representations. After the initial pretraining, fine-tuning was performed for the collected veterinary dataset using 20% of the available data. Next, a latent space exploration was performed for each model after which the encoding part of the model was fine-tuned again, this time in a supervised manner for classification. Simple Framework for Contrastive Learning of Visual Representations proved to be the most beneficial pretraining method. Therefore, it was for this method that experiments with various fine-tuning methods were carried out. We achieved a mean ROC AUC score of 0.77 and 0.66, respectively, for the laterolateral and dorsoventral projection datasets. The results show significant improvement compared to using the model without any pretraining approach.

## Introduction

Radiology is, by far, the most commonly used diagnostic imaging tool used for the investigation of thoracic pathology in cats and dogs^1^. The increasing demand for high quality veterinary services has led to an increase in the demand for fast and accurate interpretation of diagnostic images^2^. On the other hand, the reduced availability of trained veterinary radiologists struggles to meet the demand for accurate interpretation of diagnostic images^3^. In such a scenario, the role of AI-assisted tools is gaining popularity also in veterinary medicine, with an increasing number of publications on this topic^4–11^.

The ever-increasing popularity of deep learning models due to their high performance in various domains has led to an increased interest in such methods in the field of computer-aided diagnosis. A number of studies exploring the use of these modern approaches in various fields of medicine, including medical imaging have been presented^12^. Emerging solutions seek to create systems for lesion segmentation or classification in images from different imaging modalities. Some focus on detecting one specific disorder, while others attempt to annotate all of the lesions present in an image. A classification scenario in which one image can belong to several mutually non-exclusive classes is called multi-label classification. The presence of several conditions in a single examination is a common situation in medical imaging. Therefore, numerous methods dedicated to multi-label classification have already been proposed^13^. Majority of the methods use models initially pretrained on ImageNet database. The database consists of more than 14 million annotated images presenting everyday objects. The model pretraining usually improves the results but it is both computationally expensive and time-consuming since the architectures dedicated to computer vision are deeper and wider than the ones required in medical imaging. Additionally, the features presented in ImageNet database are not directly related to medical images which may reduce the impact of pretraining. Also a recently discussed topic is the carbon footprint left while training a model. Since ImageNet is a large dataset, the pretraining process takes a lot of time and memory, which results in high energy consumption. Therefore, taking into account all of the above-mentioned problems, in this work we present a different pretraining approach. We aim to improve the performance of models for classification of veterinary thoracic radiographs by self-supervised learning. We replace the standard ImageNet database by open, unlabelled inter-specie and inter-pathology XRay datasets. Using self-supervised learning not only allows to initialize the model using the same imaging modality but also does not require any expensive annotations for the pretraining.

The self-supervised setup includes variational encoders and contrastive learning frameworks. Variational encoders (VAEs) are neural networks consisting of an encoding and a decoding part, just like classical autoencoders. However, the main difference is that they aim to regularise the latent space. Thus, the encoder does not directly return a feature vector but instead the parameters of a desired distribution. As described in Ref.^14^ to balance reconstruction quality with latent space organization additional hyperparameter beta is needed. This approach allows for successful disentanglement of latent space and stable training. However, classical VAEs suffer from blurry image reconstruction. Several different solutions have been described in the literature to address this problem^15–20^. One of the most successful methods described to date is Intro-VAE^21^, which is a combination of VAE and Generative Adversarial Network (GAN). It involves training the variational encoder in an introspective manner. During training the encoder part of the network minimizes the divergence between reconstruction and input images for real data samples and maximizes it for the generated samples, while the generator model minimizes the divergence of the generated samples. The results achievable with this method are promising, but the training process is difficult. The value above which the fake sample no longer affects the loss function is a manually tuneable hyperparameter. As a result, it is difficult to stabilize the network training. The problem was addressed by authors of SoftIntro-VAE^22^. The researchers proposed the soft exponential function over the evidence lower bound (ELBO) instead of the hard threshold, which is a lot easier to optimize and more stable during training.

Another approach to self-supervised learning is contrastive learning. The idea behind this group of methods is relatively simple: the most similar images are separated by the smallest distance while the distance between all other images is being maximised. In Ref.^23^ authors proposed a Simple Framework for Contrastive Learning of Visual Representations (SimCLR). The algorithm works as follow: (i) on a batch of images data augmentation techniques are applied to create two versions of each image in a batch, (ii) the augmented images are given as an input to CNN network to obtain a 1D feature vector, (iii) a mapping with a multilayer perceptron that acts as a projection head of the encoder is performed. During training process the InfoNCE loss is used as a contrastive loss. In Ref.^24^ authors presented a mechanism for building dynamic dictionaries for contrastive learning called Momentum Contrast or MoCo. The model consist of two encoders, an encoder and momentum encoder that produce vector representations of data: queries and keys respectively. A key encoder works as a momentum-based moving average of the query encoder for maintaining consistency. During a training step positive pairs of images are constructed from queries and keys from current mini-batch meanwhile negative pairs consist of query from current mini-batch and keys from previous mini batch that are stored in queue. Another recently introduced self-supervised learning method is called Bootstrap Your Own Latent (BYOL)^25^. In this method we also create two augmented images and then we pass them as input one to the online network and the other to the target network. During the training process, the prediction outputs from the two networks are compared and the loss between them is calculated. At the same time, the target network parameters are updated so that they represent an exponential moving average of the online network parameters. The most notable difference between BYOL and other state-of-the-art methods is that it does not rely on the use of negative image pairs and, at the same time, allows comparably high results to be achieved. A solution that also does not require pairwise comparisons at all is the Swapping Assignments Between Views (SwaV) method^26^. It combines clustering with contrastive learning to make it more computationally efficient. In this approach, the two augmented versions of the image are passed to the encoders and the resulting features, rather than being compared directly, are mapped to their nearest neighbour in the set of clusters, thus producing ’codes’ that are then used to predict the features of the opposite image.

The advantage that all of the previously mentioned self-supervised learning methods bring is the use of data without the need for annotation, which is far more difficult to access than raw data. According to the National Health Service England, more than 20 million radiographs were taken in October 2020-October 2021 alone in England^27^. Given how many of these exams are performed globally each year, using some of the collected data to pretrain self-supervised learning models seems like a beneficial solution, as it eliminates the obvious domain shift that occurs when pretraining on databases such as ImageNet where the features of the images are distinctly different from those found in radiographic images. The impact of pretraining models on human X-ray images from one database and the application of these pretrained models to a different database have already been elucidated in Ref.^28^. However, the possibility of inter-species knowledge transfer has yet to be fully described. Despite anatomical differences in human and veterinary images, the features present in both types of data are similar. The use of self-supervised learning models for pretraining provides an option to improve the results obtained by the deep learning model without the need for a larger database of annotated data. This is important because the annotation process, especially for medical data that requires specialised knowledge, is a labour-intensive and therefore also time- and cost-intensive task.

### Contribution

 In this work we propose and compare self-supervised pretraining strategies on open XRay datasets to improve the performance on the downstream task related to classification of veterinary thoracic radiographs. More concretely the study: (i) compares the effectiveness of different self-supervised learning strategies pretrained on open inter-specie and inter-pathology X-Ray datasets transferred to a small, annotated veterinary dataset, (ii) explores the latent spaces for various self-supervised learning method, prior and after fine-tuning, (iii) develops a system for multi-label classification of veterinary thoracic radiographs. The study confirms that the self-supervised pretraining improves the classification accuracy without any additional data labeling.

## Results

### Database

**Figure 1:**
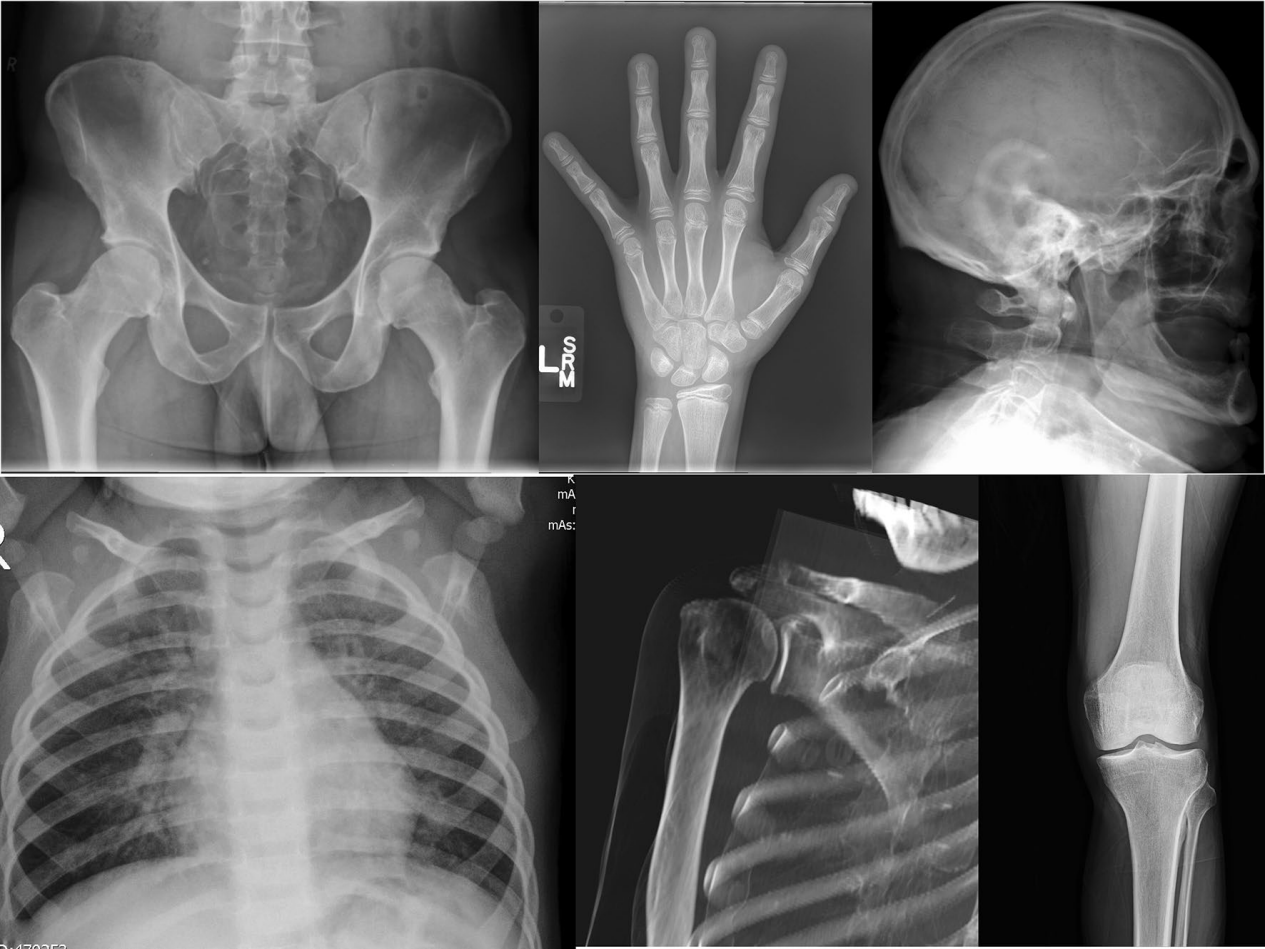
Example images from open X-Ray datasets.

The database used for pretraining the self-supervised models consists of over 600k images from 23 open XRay datasets of varying size and quality containing different anatomical structures. The increased research interest in respiratory diseases in recent years due to the joint attempts to combat the Covid-19 pandemic and its aftermath has facilitated the availability of human chest XRay images, that is why most of the collected sets contain images of this type. The detailed overview of the open datasets is provided in Table 1 and example images are presented in Fig. 1.

**Figure 2:**
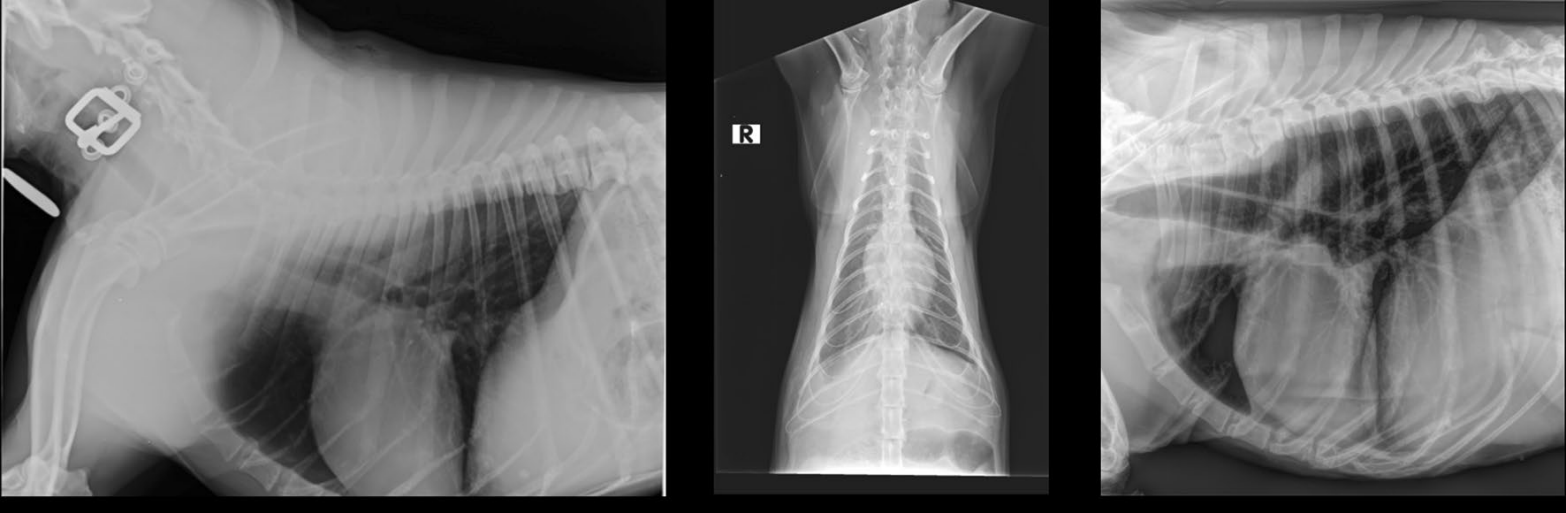
Example images from internal veterinary X-Ray dataset.

The internal, small database containing veterinary thoracic radiographs was used to fine-tune the pretrained models. It consists of 17869 annotated images they were acquired using scanners by 3 different manufacturers: Kodak, Isomedic and Fujifilm . Due to the poor quality 5453 images were excluded. The radiographs were acquired in laterolateral (LL), ventrodorsal (VD) and dorsoventral (DV) projections. The example images in different projections are presented in Fig. 2. A total of 20 different radiology findings were present in the dataset. Number of samples for each class varied significantly from 12 for edema to 2603 for cardiomegaly and 6047 for unremarkable ( this class consists of images in which no abnormalities were detected), which were the most frequent tags. The exact distribution of the number of cases for each class is shown in Table 2. Good classification results and satisfactory generalisation are not achievable if the number of samples for each class is not sufficient, which is why we selected the 9 most numerous lesions as the target for classification. The division into training, validation and test set was made with a ratio: 0.75:0.05:0.25 for each class.

### Experimental setup

The described method was implemented using Python 3.9.7 with PyTorch framework and basic, publicly available packages, including numpy, pandas, sklearn, torchvision, pydicom or kornia. The workstation on which the analysis was performed includes four NVIDIA Tesla V100 GPUs and an Intel Xeon E5-2698 v4 2.2 GHz processor.

### Self-supervised learning

**Figure 3:**
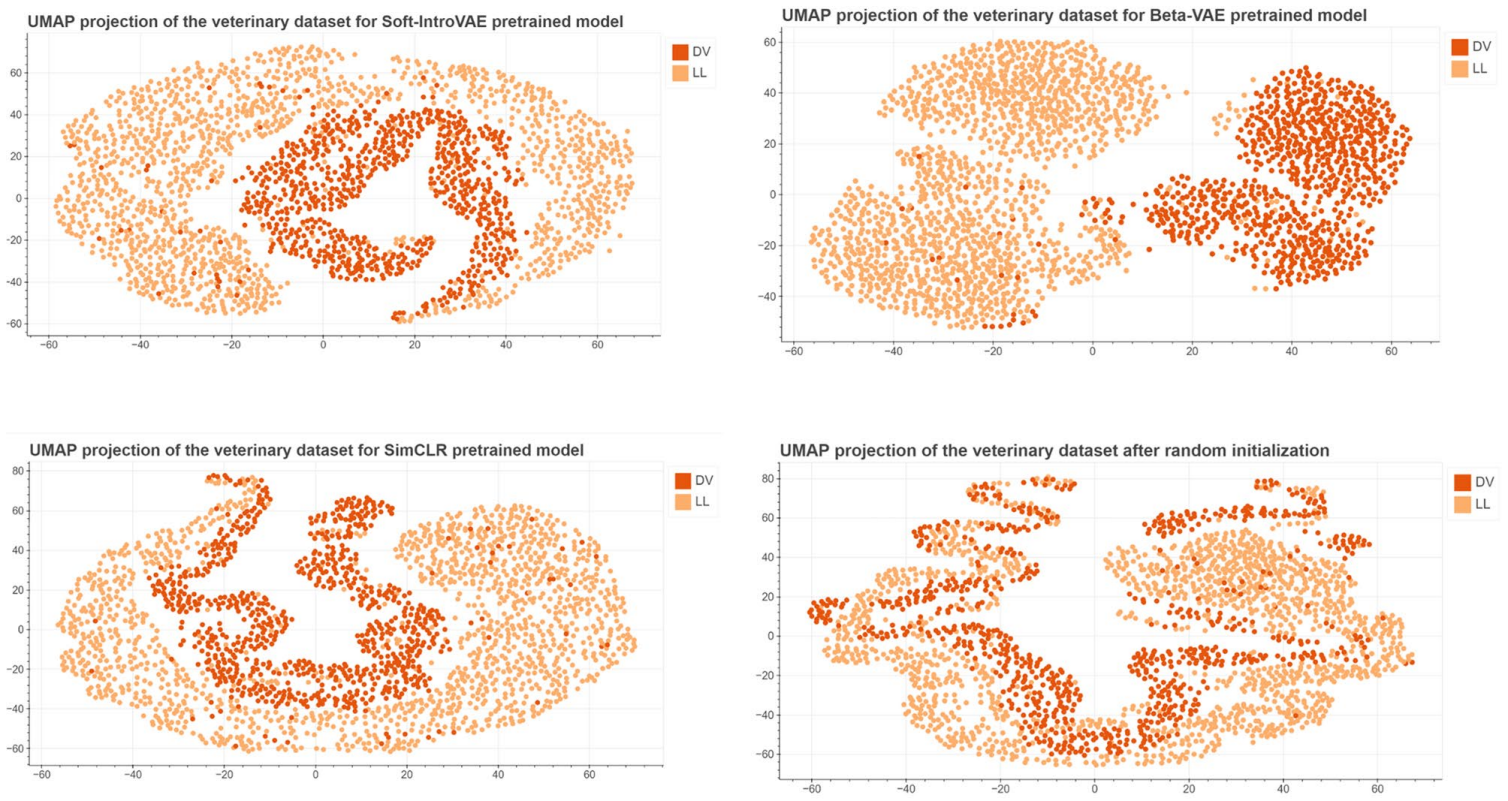
UMAP visualizations of distribution of images with different projections in the latent spaces for all used pretraining approaches: VAE, SoftIntro-VAE and SimCLR.

**Figure 4:**
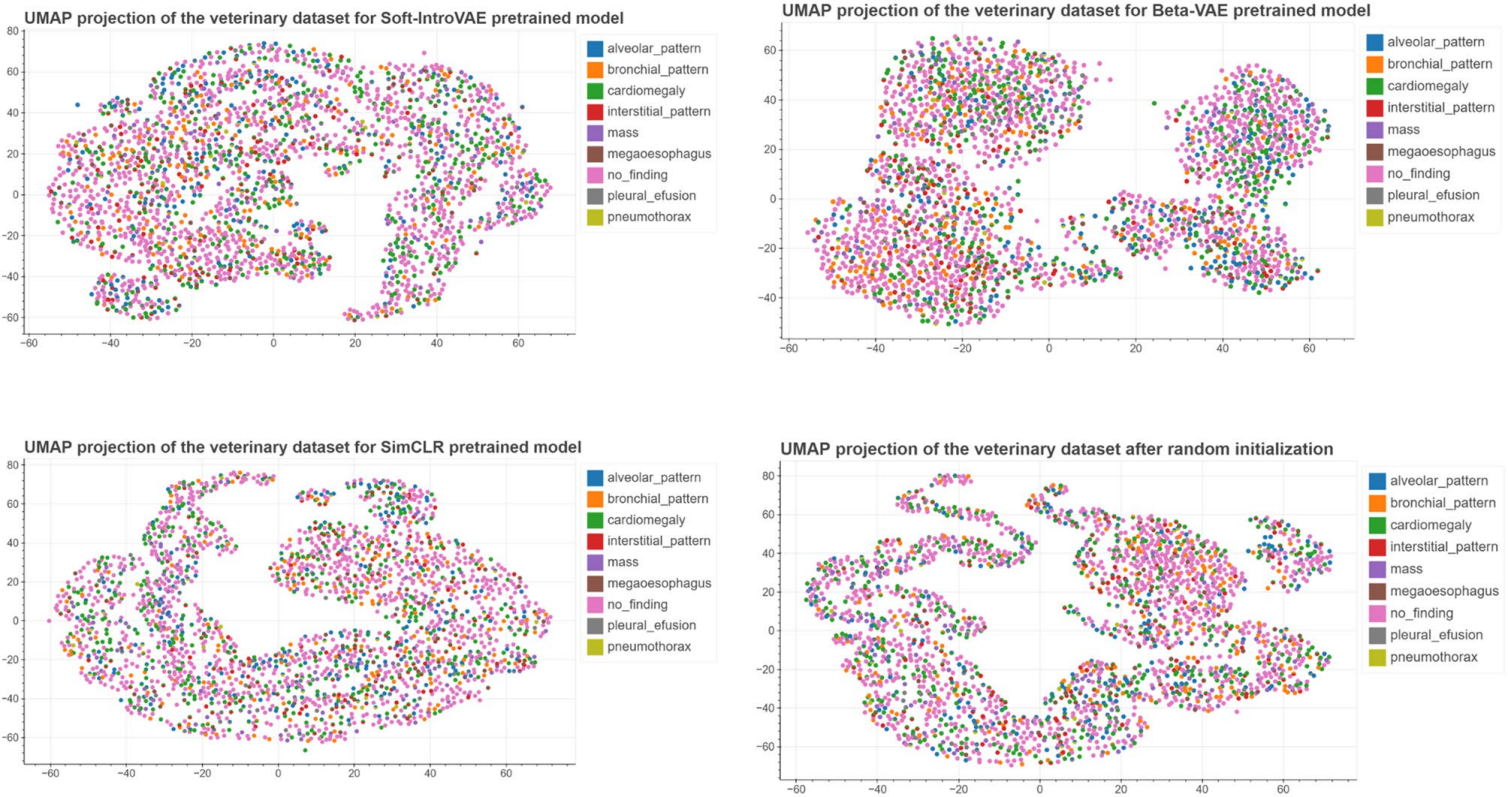
UMAP visualizations of distribution of images belonging to different classes of lesions in the latent spaces for all used pretraining approaches: VAE, SoftIntro-VAE and SimCLR.

For each of the pretrained models we used Uniform Manifold Approximation and Projection for Dimension Reduction (UMAP) to create visualizations of the latent spaces. We have noticed that images taken in ’DV’/’VD’ and ’LL’ projection are clearly separated as shown in Fig. 3. Figure 4 present distribution of images representing different classes of lesions in the latent space. The need for further finetuning for classification purposes is apparent as for neither of the models latent space is clearly organised.

**Table 1:** Pre-training database overview with the names of the individual data sets, a brief description and the number of images it contains.

Database name	Description	Number of images
PadChest^29^	Annotated high resolution chest x-ray images	160,000
MURA^30^	Large dataset of musculoskeletal radiographs	40,561
ChestX-ray14^31^	Frontal orientation chest X-Ray images	112,120
IRMA^32^	Dataset containing anonymised X-ray imagest that reflect different age groups, genders, exposure positions and pathologies.	14,410
TCGA-BLCA^33^	The Cancer Genome Atlas Urothelial Bladder Carcinoma X-Ray images data	74
COVID-19-NY-SBU^34^	Collection of chest X-Ray images from Covid-19 patients	902
CPTAC-PDA^35^	Images from the Clinical Proteomic Tumor Analysis Consortium containing imaging data of patients with pancreatic ductal adenocarcinoma	7
CPTAC-LSCC^36^	Collection of chest X-Ray images from Clinical Proteomic Tumor Analysis Consortium containng images with lung squamous cell carcinoma	2
MIDRC-RICORD-1c^37^	Imaging data from patients who suffered from Covid-19	296
COVID-19-AR^38^	Radiology imaging from Covid-19 positive population	192
CPTAC-UCEC^39^	Imaging data from National Cancer Institute’s Clinical Proteomic Tumor Analysis Consortium taken from patient with uterine corpus endometrial carcinoma.	1
ACRIN-NSCLC-FDG-PET^40^	Data from multicenter clinical trail hold by American College of Radiology Imaging Network	20
LIDC-IDRI^41^	Dataset collected to help with developing computer-aided methods of cancer detection	109
Chest X-Ray Images (Pneumonia)^42^	Dataset of chest X-rays from both healthy and pneumonia patients	5856
UNIFESP X-ray^43^	DICOM image database containing radiographs displaying 22 different parts of the body	2480
Aseptic Loose Hip Implant X-Ray^44^	A collection of radiographs including at least a stem and a cup of the hip implant collected from available sources such as medical journals and radiology websites	200
Shoulder Implant X-Ray^45, 46^	X-Ray images of patients with shoulder implants collected at Biomedical Image and Data Analysis Lab in San Francisco State University	597
Osteoporosis Knee X-ray^47^	Collection of radiographs of the knee of healthy patients as well as those with osteoporosis	372
Shoulder X-ray	Shoulders X-Ray images from patients at Sun Yat-sen Memorial Hospital of Sun Yat-sen University, Guangzhou	1049
COVID-19 Radiography Database^48, 49^	Chest X-ray images database consisted of covid-19 positive cases as well as pneumonia and healthy lung images	33,920
Bone Age^50^	Dataset from Stanford University and the University of Colorado consisting of hand radiographs	14,236
CheXpert^51^	Large dataset with presence of 14 different radiology observations	224,316
Radiographic Dataset for VHS determination learning process^52^	Dataset of canine laterolateral thoracic radiographs	156
	Total number of images	511,876

**Table 2:** Veterinary data overview.

Radiographic finding	Number of samples
Cardiomegaly	2603
Alveolar pattern	1818
Bronchial pattern	1308
Interstitial pattern	997
Pleural efusion	893
Mass	735
Pneumothorax	326
Megaoesophagus	230
Unremarkable	6047
Pneumoderma	121
Foreign body	114
Hernia	99
Suture	76
Fracture	64
Pneumomediastinum	47
Tracheal collapse	44
Edema	12

### Classification results

**Figure 5:**
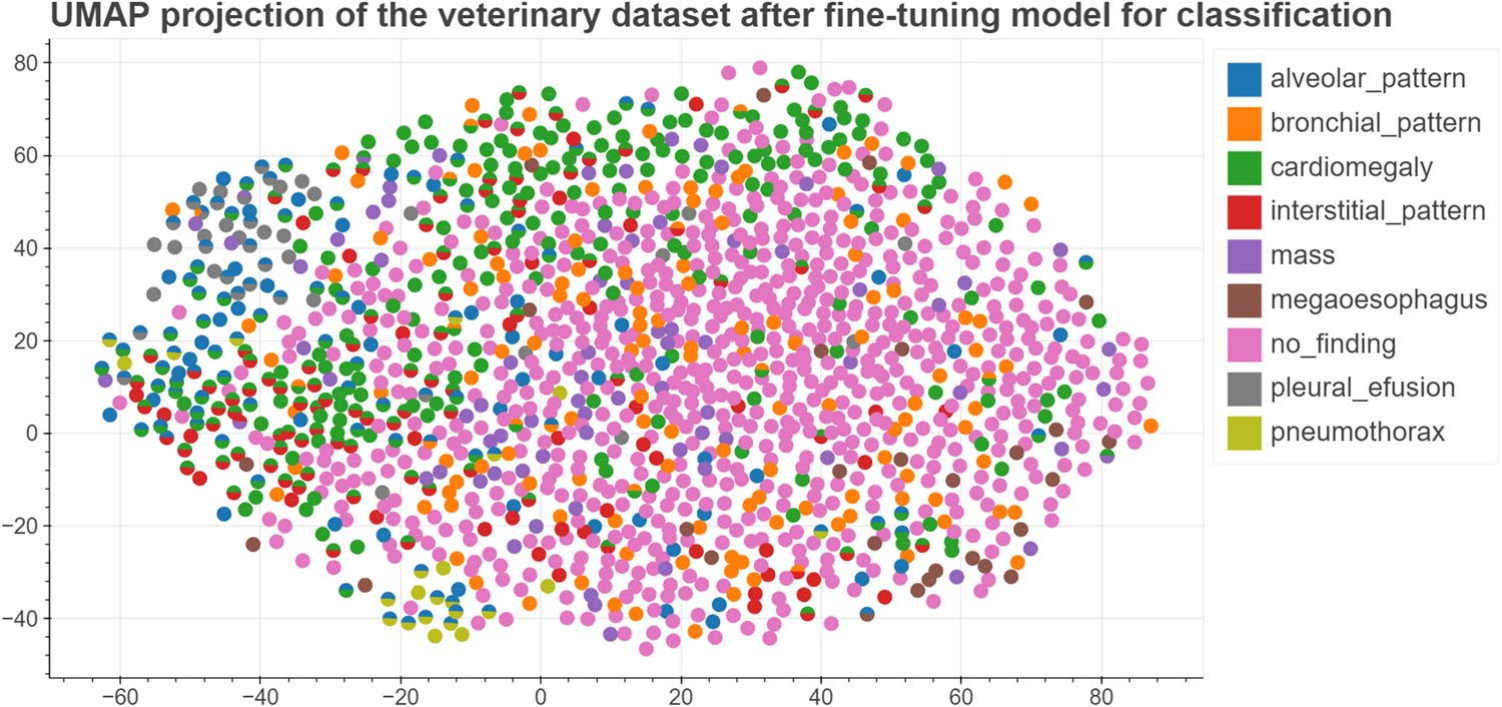
Latent space after fine-tuning the model for classification.

**Figure 6:**
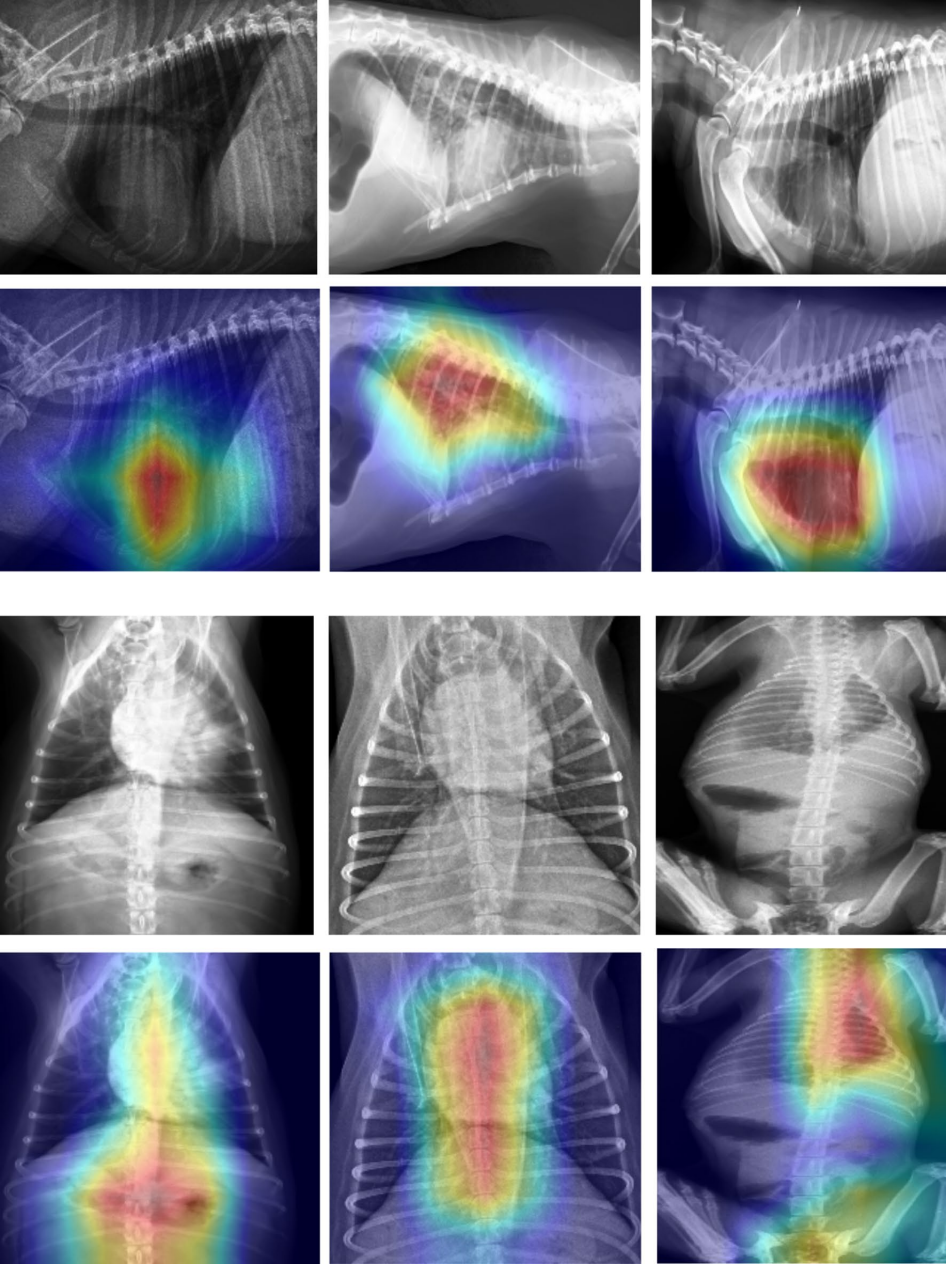
GradCAM image examples alongside original images. The first two rows show (from left) cardiomegaly, alveolar pattern, and bronchial pattern, while the last two rows (from left) show mass, megaesophagus, and pleural effusion.

As mentioned above, the radiological images are clearly separated in the latent space in terms of projection. Given this as well as the significant difference in image number in the ’DV’ and ’LL’ projections, the model evaluation was carried out separately for image sets belonging to the two projection classes. In order to assess which of the pre-training approaches brings the most benefit in terms of using an encoder for classification, we performed a simple test: in each of the models we unfroze last layer and added additional linear layer serving as a classifier. Comparison of classification results obtained with different pretraining approaches is presented in Table 3. The best results were achieved with a model pretrained using contrastive learning framework, providing mean ROC AUC 0.74 for ’LL’ projection dataset and 0.66 for ’DV’ projection. In the next step we proceeded to try to get a better result using other finetuning methods only for the best of the models. Results of all conducted studies are presented in Table 4. The best results for both of the datasets were achieved while training model with 9 binary classifiers (one for each class) and last layer unfrozen. This method allowed us to obtain significant improvement in obtained results when compared to model without pretraining. Figure 5 present distribution of images representing different classes of lesions in the latent space after fine-tuning process, the distribution of images in the latent spaces for each class separately can be found as Supplementary Figs. S1–S9. Detailed results of classification consisting of ROC AUC score alongside sensitivity, specificity, PLR and NLR for each class in DV and LL projection datasets are presented in Tables 5 and 6. Additionally, to aid in the interpretation of the biological relevance of the obtained results, Fig. 6 showcases GradCAM image examples of different classes from both projections. It can be observed that the activation maps are spatially correlated with the classified lesions.

**Table 3:** Comparison of different pretraining approaches for classification of lesions belonging to 9 lesion classes.

Model	AUC	
	Dorsoventral	Laterolateral
Beta-VAE	0.61	0.71
Soft-IntroVAE	0.64	0.73
**SimCLR**	**0.66**	**0.74**
Baseline	0.59	0.69

Highest achieved scores are in bold.

**Table 4:** Comparison of different finetuning approaches.

Model	AUC	
	DV	LL
1 classifier		
Last layer unfrozen	0.66	0.74
Last two layers unfrozen	0.65	0.76
All layer frozen with additional linear layer	0.63	0.72
9 binary classifiers		
** Last layer unfrozen**	**0.66**	**0.77**
Last two layers unfrozen	0.64	0.76
All layer frozen with additional linear layer	0.61	0.71
SVM classification	0.63	0.70

Highest achieved scores are in bold.

**Table 5:** Results of classification with the best model (model with 9 binary classifiers and last layer unfrozen) for each class on dataset with ’LL’ projection.

Lesion	AUC	Sensitivity	Specificity	PLR	NLR
Cardiomegaly	0.77	0.78	0.58	1.86	0.37
Alveolar pattern	0.78	0.74	0.70	2.48	0.38
Bronchial pattern	0.63	0.68	0.50	1.36	0.64
Interstitial pattern	0.71	0.76	0.59	1.83	0.42
**Pleural efusion**	**0.93**	**0.96**	**0.64**	**2.63**	**0.07**
Mass	0.65	0.65	0.60	1.62	0.59
**Pneumothorax**	**0.91**	**0.77**	**0.85**	**5.14**	**0.27**
Megaoesophagus	0.70	0.54	0.75	2.12	0.62
Unremarkable	0.73	0.83	0.50	1.66	0.34

Highest achieved scores are in bold.

**Table 6:** Results of classification with the best model (model with 9 binary classifiers and last layer unfrozen) for each class on dataset with dorsoventral (’DV’) projection.

Lesion	AUC	Sensitivity	Specificity	PLR	NLR
Cardiomegaly	0.58	0.81	0.28	1.02	0.93
Alveolar pattern	0.66	0.80	0.41	1.35	0.49
Bronchial pattern	0.54	0.82	0.27	1.50	0.40
Interstitial pattern	0.68	0.87	0.45	2.37	0.21
**Pleural efusion**	**0.72**	**0.69**	**0.63**	**1.07**	**0.88**
Mass	0.50	0.60	0.35	1.57	1.12
Pneumothorax	0.70	0.63	0.61	1.56	0.60
**Megaoesophagus**	**0.73**	**0.82**	**0.59**	**2.02**	**0.30**
Unremarkable	0.61	0.92	0.21	1.15	0.41

Highest achieved scores are in bold.

## Discussion

An important consequence of this article is that it demonstrates how, thanks to approaches that do not require human labels such as self-supervised learning, it is possible to benefit from inter-specie and inter-pathology data to improve deep learning models for medical applications. This result paves the way to models that could benefit of increasingly available data on different species, allowing to create increasingly realiable models and potentially shifting the paradigm from classification to unsupervised inter-specie knowledge extraction problems.

Achieved results suggest that features learned from images presenting particular specie can be used to improve classification result for another specie. These clinical insights could potentially have significant repercussions. One of the potential applications of the presented self-supervised learning model could be in the diagnosis of those diseases that are rare in humans but have a relatively higher incidence in animals^53^. By pre-training the model on a multi-species dataset and then fine-tuning it on a much smaller human dataset, it could be used to accurately diagnose diseases that some dog breeds are more prone to, such as histiocytic sarcoma in Bernese mountain dogs, gliomas in boxers and juvenile dilated cardiomyopathy in Portuguese water dogs. The potential to employ a cross-species strategy could revolutionize the relationship between human and animal medical care, thereby progressing towards a One-Health medicine.

Using more balanced dataset for fine-tuning self-supervised model would probably lead to achieving better performance, as we can see significantly difference in results for ’DV’ and ’LL’ projection datasets. Also, to improve the performance of the presented approach, other encoder architectures can be implemented, for example based on Vision Transformer (ViT) instead of CNN, as literature shows its promising results in image recognition tasks^54^.

## Methods

### Data acquisition

Open, universally available databases containing human radiographs were used for the pretraining part of presented solution. Fine-tuning, on the other hand, was carried out using an internal veterinary dataset. The methods used to create the database for this study are described elsewhere^5^. Briefly, all the radiographs included in the database were evaluated by three experienced veterinary radiologist (AZ, TB, and SB), for several different radiographic abnormalities (alveolar pattern, interstitial pattern, bronchial pattern, mass, cardiomegaly, pleural effusion, pneumothorax, hernia, megaoesophagus, fracture, pneumomediastinum, tracheal collapse). If no radiographic findings were evident, the image was classified as unremarkable. All the tags on the images were applied following a consensus discussion. Image quality was also assessed and only technically correct images were included in the database.

### Overview

**Figure 7:**
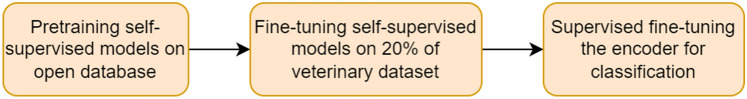
Brief overview of proposed approach.

The first step in image preprocessing was to resize images to equal size, in our case: 224x224 pixels. The images were then normalised so that the intensity was in a range of 0-1. In the next step three different self-supervised architectures were pretrained using unlabelled data from open database. For this purpose, the following were selected: BetaVAE, Soft-IntroVAE and SimCLR. Each of the models was later fine-tuned using 20% of available annotated veterinary data. After fine-tuning latent space exploration using UMAP was carried out. In the latent space, a clear division was evident in terms of features such as image projection, but additional fine tuning was needed to obtain better division results for lesions visible on radiographs. Due to the possibility of several lesions being present on a single image, a multilabel classification approach had to be used. In the classification step the encoding part of the model was used which was extended with linear layer to classify features extracted by the encoder. Three approaches were compared: the introduction of one additional linear layer with 9 outputs, the addition of 9 binary classifiers, one for each lesion type, and the use of an SVM as a classifier. A brief summary of the pipeline is presented in Fig. 7.

### Self-supervised learning

**Figure 8:**
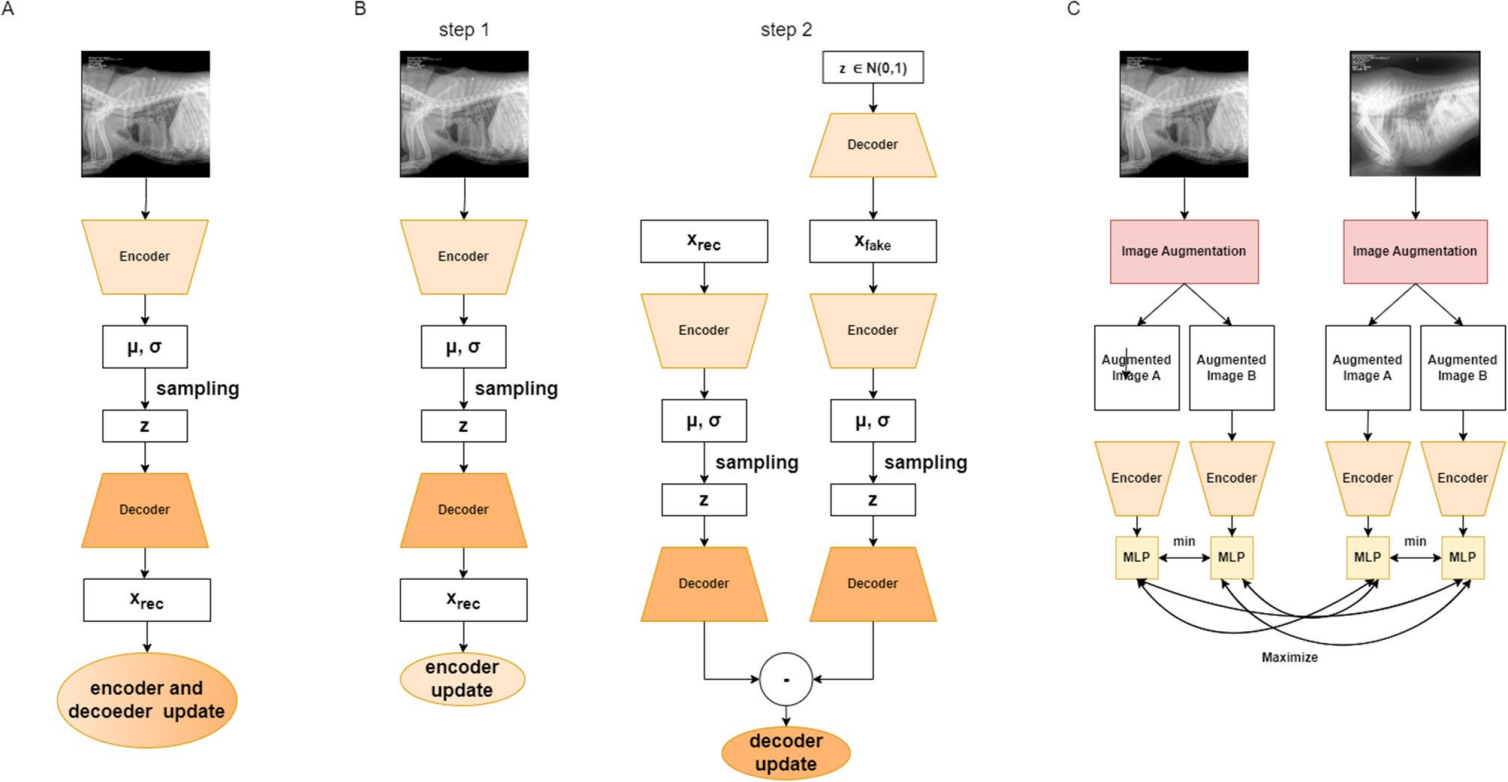
Block diagrams of self-supervised pretraining techinques.

For all pretraining approaches we used the same network architecture. The encoder was built with 5 residual blocks with 64, 128, 256, 512 and 512 channels. Additionally for VAE-based approaches the same setting was used as a decoder part. For Beta Variational Autoencoder training beta parameter was set to 0.01 and L1 loss was chosen as a cost function. Soft-Intro VAE was trained using MSE loss function with beta neg equal to 256. During SimCLR Info Nce Loss was minimized. For all of the approaches batch size was set to 32. A comparison of how each approach was trained is presented in Fig. 8.

### Classification fine-tuning

For fine-tuning purposes, we conducted a number of tests with different modifications of the classification model. However, firstly, in order to establish a baseline for comparing the different approaches, we used the encoder network without any pre-training to see what results it was able to achieve in classifying 9 selected lesions. As the dataset was highly imbalanced to increase the effectiveness of the model we implemented a balancing sampler so that the representation of each class was closer to equal. In addition, to improve performance on the training set, we also used image augmentation techniques such as vertical and horizontal rotation as well as brightness, contrast and saturation augmentations. We used these steps for each finetuning method tested. We tested 4 different fine-tuning approaches including: classification with unfreezing last layer of the model, unfreezing last two layers of the model, adding additional linear layer to model with all weights frozen. Tests were performed for one classification layer with 9 outputs corresponding to each lesion as well as with 9 binary classifiers one for each lesion. For all the approaches considered, the initial value of the learning rate was set to 0.01. A lambda learning rate scheduler was employed, causing the learning rate to decrease by a decay rate (set to 0.95) raised to the power of the current epoch’s number. Additionally, we also performed classification using features extracted with pretrained model with all weights frozen and Support Vector Machine (SVM).

#### Result analysis

We evaluated the performance of the individual classification methods on both the data set containing the ’LL’ projection and the data set containing the ’DV’ projection by calculating values such as area under the receiver operating characteristic curve (AUC), sensitivity, specificity, positive likelihood ratio (PLR) and negative likelihood ratio (NLR) according to the formulas in Eqs. (1), (2), (3) and (4).

1 $$\begin{aligned} Sensitivity= \frac{true\,positive}{true\,positive + false\,negative} \end{aligned}$$

2 $$\begin{aligned} Specificity= \frac{true\,negative}{true\,negative + false\,positive} \end{aligned}$$

3 $$\begin{aligned} PLR= \frac{sensitivity}{ 1 - specificity} \end{aligned}$$

4 $$\begin{aligned} NLR= \frac{ 1 - sensitivity}{specificity} \end{aligned}$$

## Conclusions

The impact of pretraining self-supervised models on the accuracy of automatic multi-label classification of lesions in veterinary radiographs was studied. The detection accuracy for each class was higher for the model that had been pretrained, compared to the model without pretraining. Further studies, with a larger dataset and possibly a different encoder architecture, could lead to the development of a system that can aid veterinarians in their diagnosis.

### Supplementary Information

Supplementary Figures.

## Data Availability

The datasets generated and/or analysed during the current study are not publicly available due to privacy restrictions but are available from the corresponding author on reasonable request.
