# Supplementary material for: Improving the classification of veterinary thoracic radiographs through inter-species and inter-pathology self-supervised pre-training of deep learning models
Source: Sci Rep. 2023 Nov 9;13:19518. doi: 10.1038/s41598-023-46345-z (PMC10636209; doi:10.1038/s41598-023-46345-z)
Supplement: Supplementary file 1 — Supplementary Figures. [file 41598_2023_46345_MOESM1_ESM.pdf]

Supplementary Material

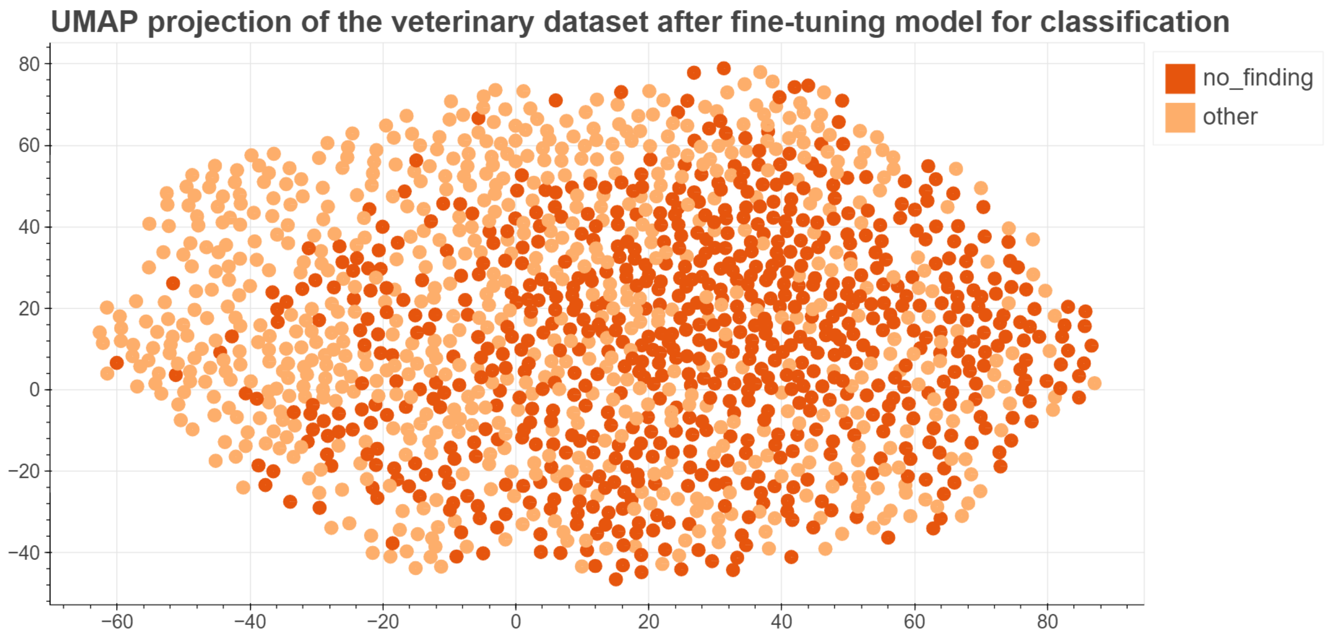

Supplementary Figure S1. UMAP visualizations of distribution of images from 'no finding' class in the latent spaces after fine-tuning model for classification. All of images represent only one class.

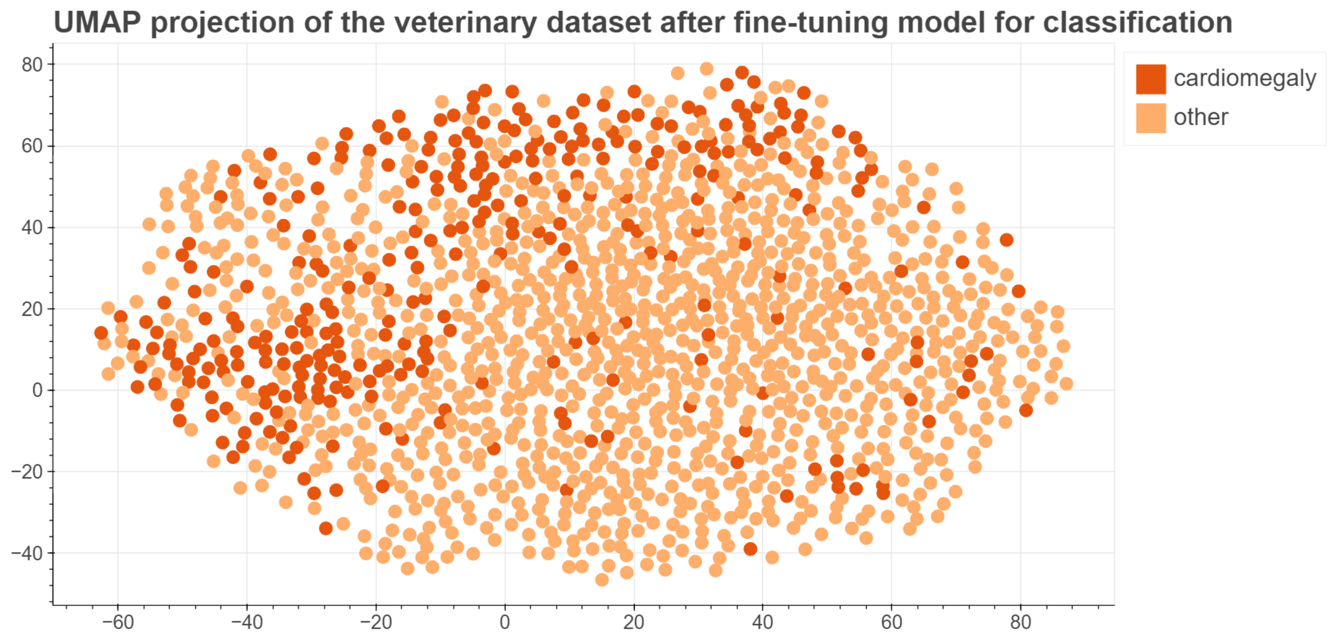

Supplementary Figure S2. UMAP visualizations of distribution of images from 'cardiomegaly' class in the latent spaces after fine-tuning model for classification. For this class 64% of the images belong to more than one class.

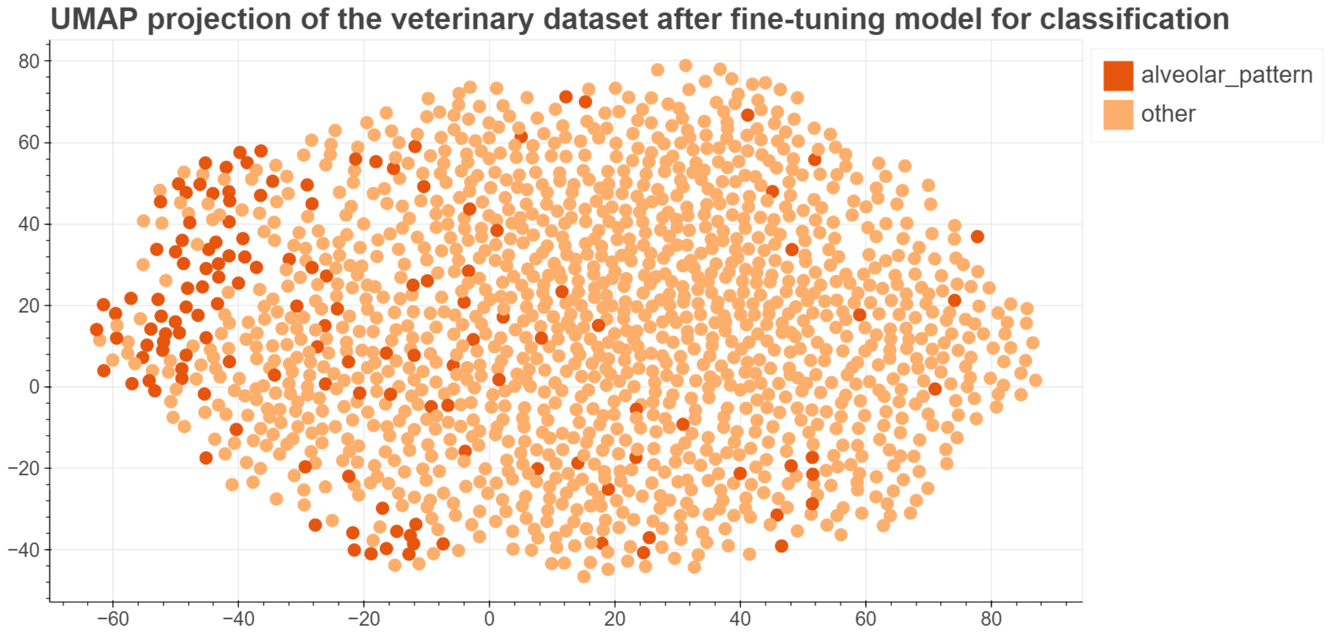

Supplementary Figure S3. UMAP visualizations of distribution of images from ’alveolar pattern’ class in the latent spaces after fine-tuning model for classification. For this class 79% of the images belong to more than one class.

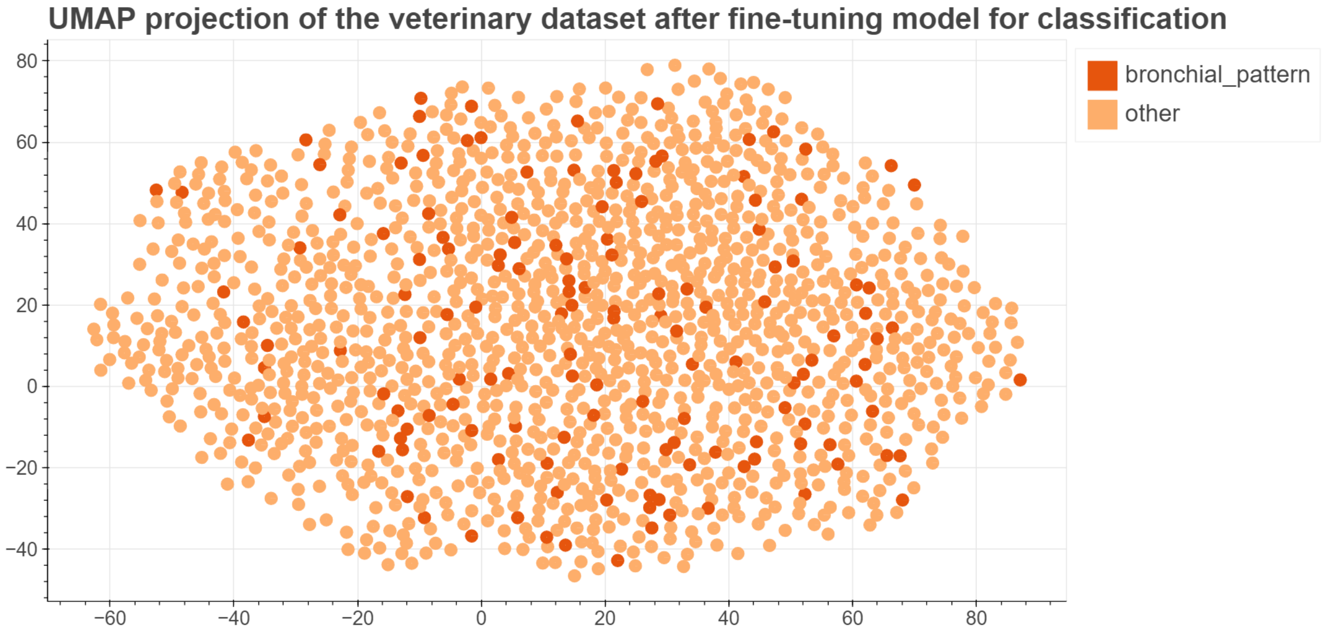

Supplementary Figure S4. UMAP visualizations of distribution of images from ’bronchial pattern’ class in the latent spaces after fine-tuning model for classification. For this class 18% of the images belong to more than one class.

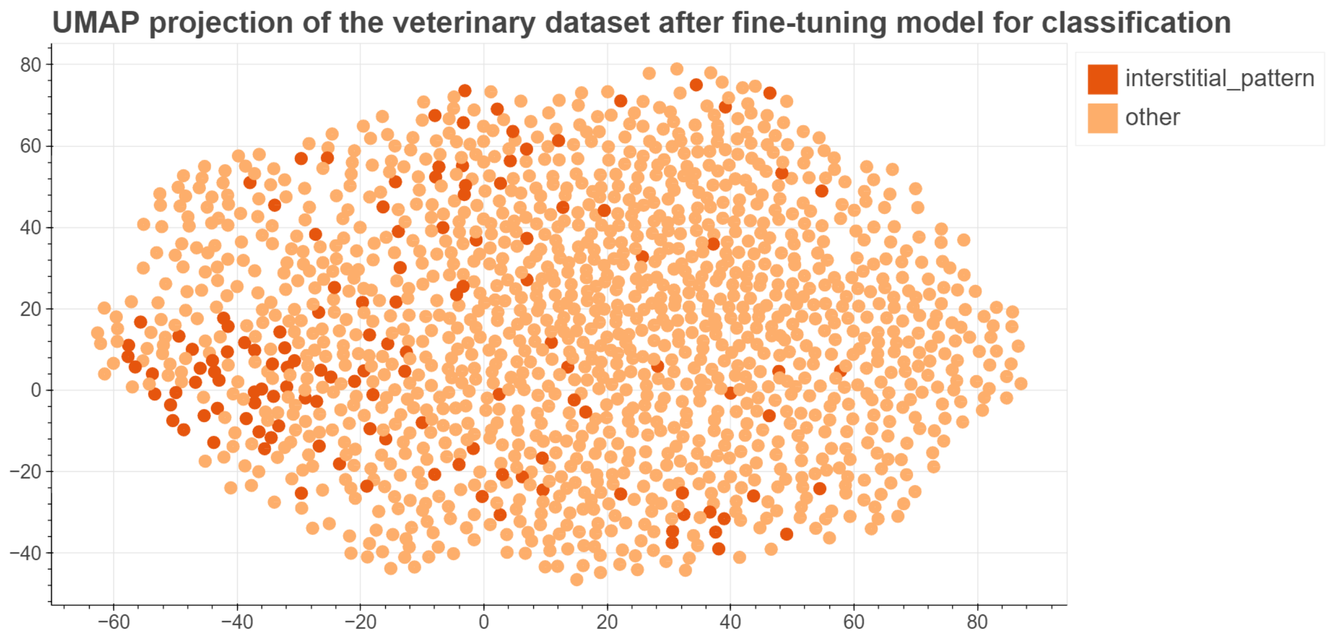

Supplementary Figure S5. UMAP visualizations of distribution of images from ’interstitial pattern’ class in the latent spaces after fine-tuning model for classification. For this class 82% of the images belong to more than one class.

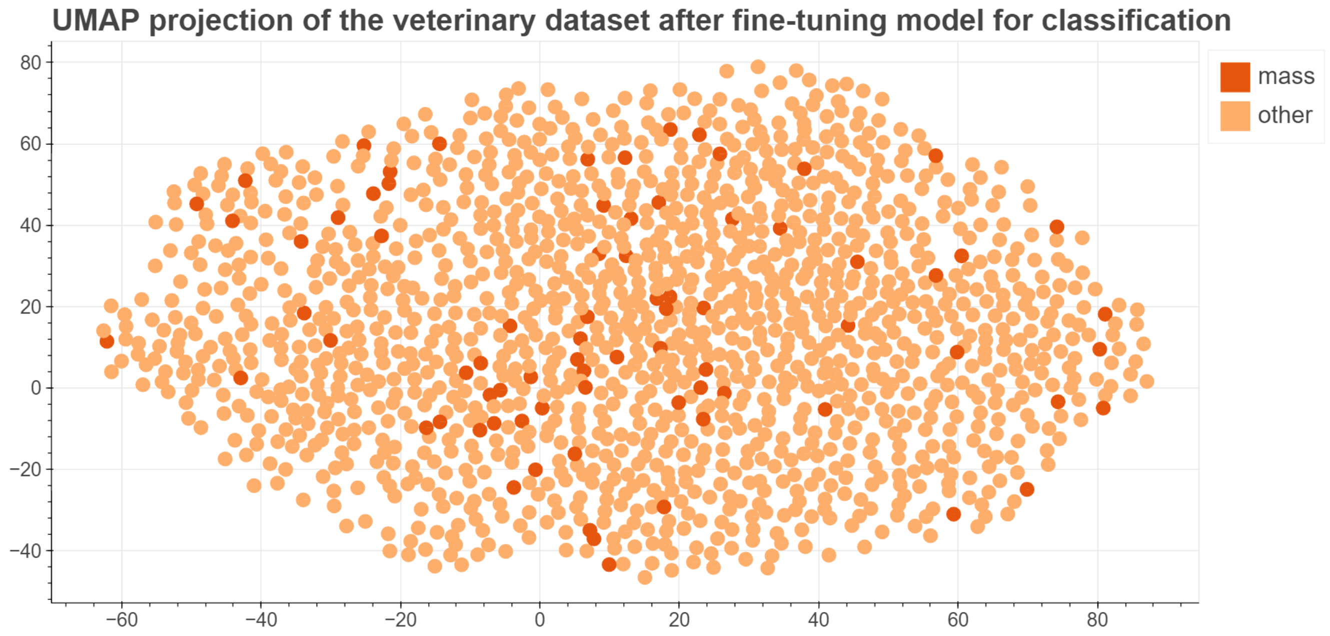

Supplementary Figure S6. UMAP visualizations of distribution of images from ’mass’ class in the latent spaces after fine-tuning model for classification. For this class 10% of the images belong to more than one class.

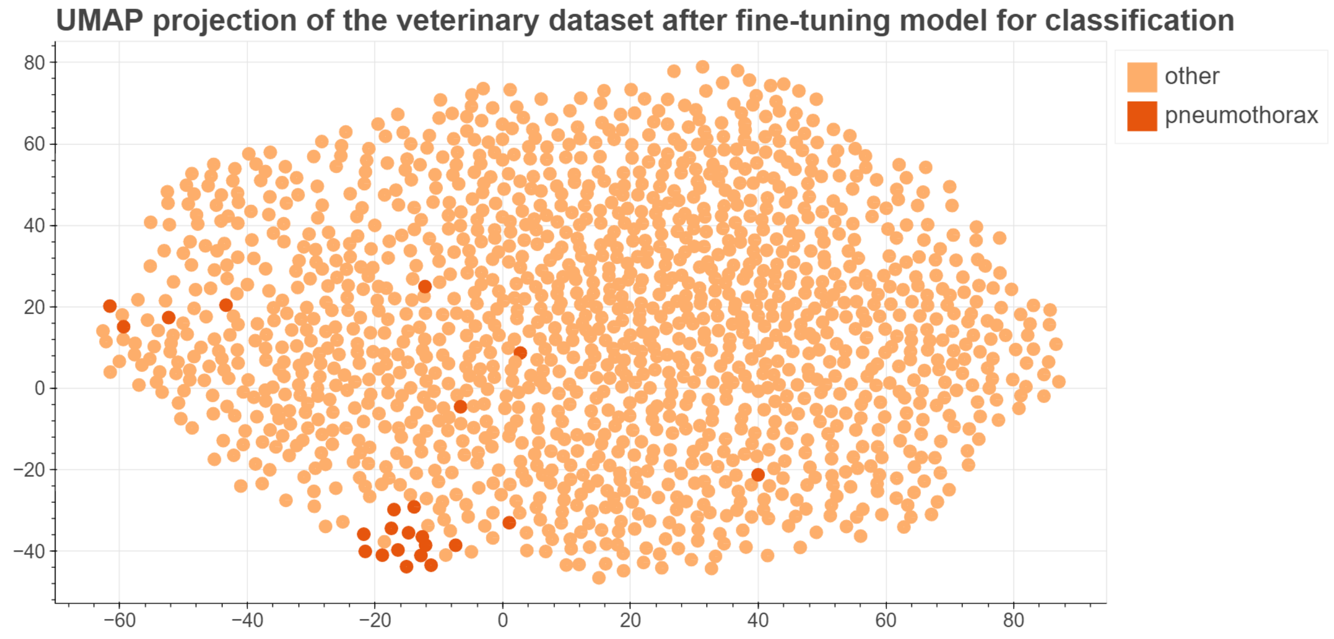

Supplementary Figure S7. UMAP visualizations of distribution of images from 'pneumothorax' class in the latent spaces after fine-tuning model for classification. For this class 82% of the images belong to more than one class.

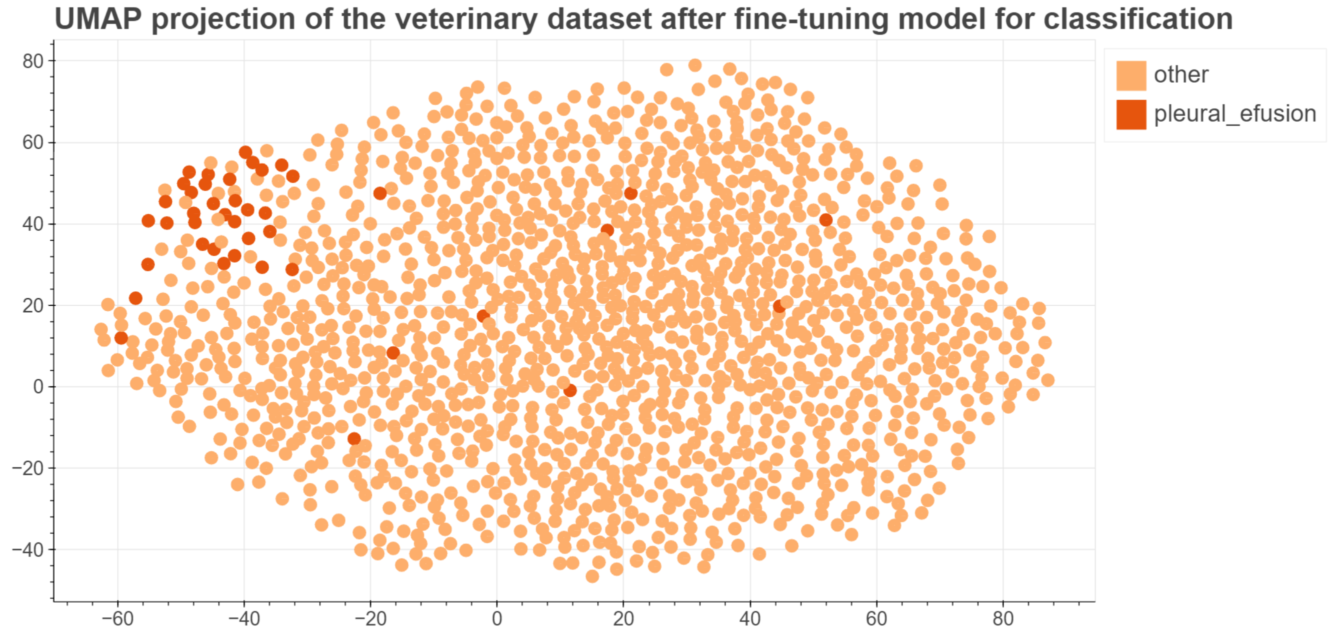

Supplementary Figure S8. UMAP visualizations of distribution of images from 'pleural efusion' class in the latent spaces after fine-tuning model for classification. For this class 61% of the images belong to more than one class.

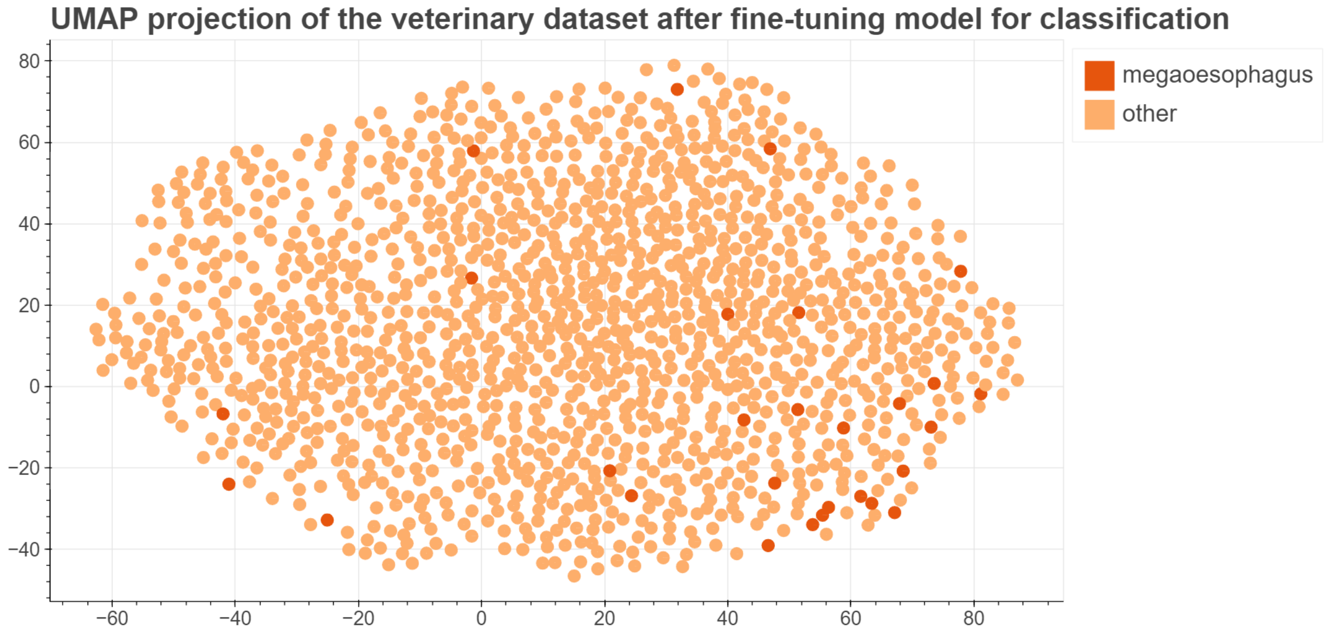

Supplementary Figure S9. UMAP visualizations of distribution of images from 'megaoesophagus' class in the latent spaces after fine-tuning model for classification. For this class 7% of the images belong to more than one class.
